# A Handbook for Nurses

**Published:** 1900-06

**Authors:** 


**A Handbook for Nurses.** By J. K. Watson, M.D. Pp. xii. 413. London: The Scientific Press, Ld. 1899.

The object of this book is to combine in one volume anatomy, physiology, medicine, surgery, midwifery, and general nursing. To do this in a book of this size is an ambitious task. There is no doubt that it is absurd for a nurse to attempt to learn what she may want to know of medical subjects from the ordinary text-books. This handbook covers in each branch most of the principles, and will supply a want which has been often felt by nurses. The details of actual nursing have necessarily had to be put rather shortly; but in conjunction with some small text-book purely on this subject, a nurse will have quite sufficient literature for ordinary purposes.

About the actual facts in the book there is little to say. Dr. Watson seems to have avoided putting forward his own opinions, but to have gathered the most prevailing ones and to have stated them very clearly.

The diagrams and tables are well selected, and a very careful index has been compiled. Anyone who has attempted to reduce knowledge to a small space in print will understand that a great amount of care and patience has been bestowed here, and the result is a work which is sure to be much read.

